# Advances in Interferometric Synthetic Aperture Radar Technology and Systems and Recent Advances in Chinese SAR Missions

**DOI:** 10.3390/s25154616

**Published:** 2025-07-25

**Authors:** Qingjun Zhang, Huangjiang Fan, Yuxiao Qin, Yashi Zhou

**Affiliations:** 1 Institute of Remote Sensing Satellite, China Academy of Space Technology, Beijing 100094, China; ztzhangqj@163.com (Q.Z.); 18811333238@163.com (H.F.); 2 School of Electronic and Information, Northwestern Polytechnical University, Xi’an 710072, China; yuxiao.qin@nwpu.edu.cn

**Keywords:** InSAR, D-InSAR, single-pass, repeat-pass

## Abstract

With advancements in radar sensors, communications, and computer technologies, alongside an increasing number of ground observation tasks, Synthetic Aperture Radar (SAR) remote sensing is transitioning from being theory and technology-driven to being application-demand-driven. Since the late 1960s, Interferometric Synthetic Aperture Radar (InSAR) theories and techniques have continued to develop. They have been applied significantly in various fields, such as in the generation of global topography maps, monitoring of ground deformation, marine observations, and disaster reduction efforts. This article classifies InSAR into repeated-pass interference and single-pass interference. Repeated-pass interference mainly includes D-InSAR, PS-InSAR and SBAS-InSAR. Single-pass interference mainly includes CT-InSAR and AT-InSAR. Recently, China has made significant progress in the field of SAR satellite development, successfully launching several satellites equipped with interferometric measurement capabilities. These advancements have driven the evolution of spaceborne InSAR systems from single-frequency to multi-frequency, from low Earth orbit to higher orbits, and from single-platform to multi-platform configurations. These advancements have supported high precision and high-temporal-resolution land observation, and promoted the broader application of InSAR technology in disaster early warning, ecological monitoring, and infrastructure safety.

## 1. Introduction

Interferometric synthetic aperture radar (InSAR) measurement technology, an active quantitative microwave remote sensing technique developed over the past half-century, has been verified as an important technical means for Earth observation [1,2,3,4,5,6,7,8,9]. It originated from “Young’s double-slit interference experiment”, proposed by Thomas Young in 1801. By leveraging both amplitude and phase information from SAR imagery for interferometric processing, InSAR plays a decisive role in global topographic mapping, marine hydrological observation, disaster prevention and mitigation, etc., featuring high precision and wide coverage.

Rogers and Ingalls [10] were the first to apply interferometry techniques to terrain observation of Venus and the Moon. In 1974, L.C. Graham [11] proposed the technical principle of using InSAR technology for topographic surveys, demonstrating the feasibility of InSAR for topographic surveying for the first time through X-band airborne radar implementations. Subsequently, the National Aeronautics and Space Administration (NASA)’s Jet Propulsion Laboratory (JPL) in the United States conducted the first experimental research on the airborne InSAR three-dimensional reconstruction of terrain using airborne side-looking radar, obtaining a digital elevation model (DEM) of San Francisco [12]. In 1988, Goldstein et al. [12] processed SEASAT satellite observation data using InSAR technology, and the topographic maps of Death Valley obtained by them were consistent with the results published by the United States Geological Survey.

**Figure 1:**
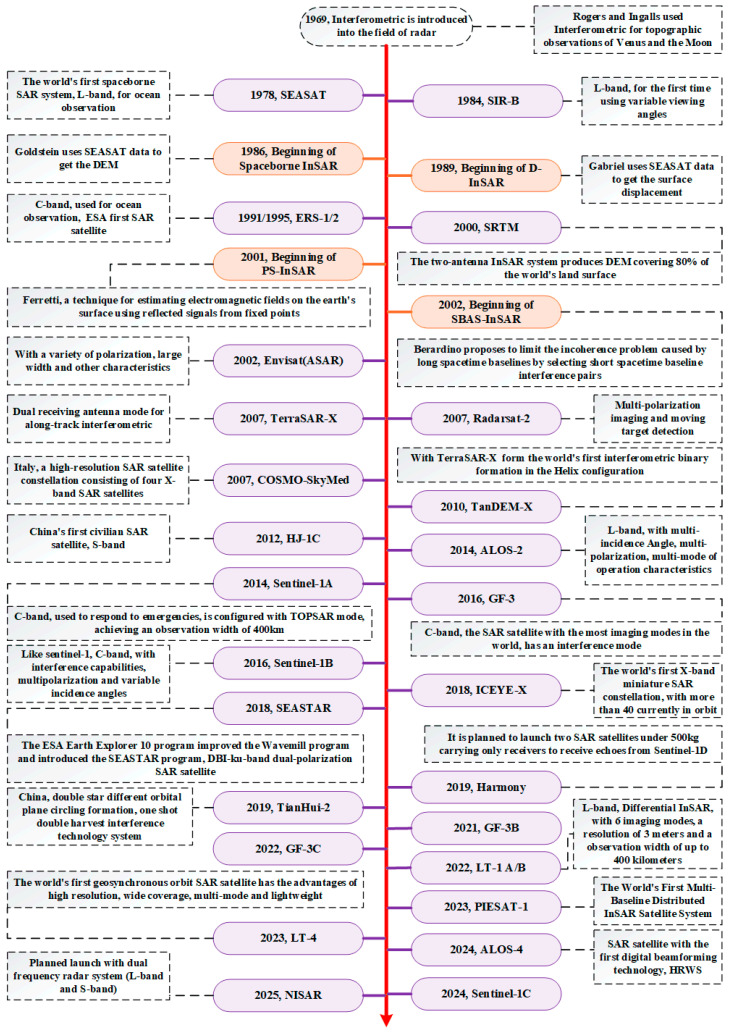
Development history of the world’s major space-borne InSAR systems and technologies.

With the launch and operation of numerous of SAR satellites, scholars conducted a large number of studies on repeated-pass InSAR technology, effectively promoting the rapid development of related theoretical methods and application technologies. Due to the serious atmospheric delay differences, temporal decorrelation, and low baseline measurement accuracy between multiple passes, repeated-pass InSAR is primarily limited to surface deformation monitoring and cannot satisfy digital elevation accuracy requirements [13]. To address these constraints, single-pass interferometric SAR can simultaneously obtain multiple coherent SAR images. For instance, the pioneering Shuttle Radar Topography Mission (SRTM) generated DEM within the range from 56° south latitude to 60° north latitude, which conformed to the Digital Terrain Elevation Data level 2 (DTED-2) standard [14]. The development of InSAR has gone through the process of “ground detection radar–imaging synthetic aperture radar–synthetic aperture radar interferometry”, which also indicates that InSAR is the integration of synthetic aperture radar remote sensing imaging and electromagnetic wave interference technologies. Key milestones in onboard InSAR systems and technical development history are shown in Figure 1.

China’s spaceborne SAR missions have continuously advanced and accelerated, gradually forming a relatively complete technical system and application framework. Since the launch of HuanJing-1C (HJ-1C), the HJ-1C satellite equipped with an S-band SAR system in 2012, China has officially entered the era of spaceborne SAR. The GaoFen-3 (GF-3) satellite, launched in 2016, is China’s first C-band fully polarimetric SAR imaging satellite, with a resolution reaching 1 m [15,16,17], providing crucial support for high-resolution imaging and surface deformation monitoring of the land and ocean [18,19]. Subsequently, the LuTan-1 (LT-1), the world’s first L-band distributed-formation multi-polarization InSAR altimetry satellite system, achieved meter-level ground observation resolution and successfully carried out China’s first on-orbit bistatic interferometric observation, marking a key breakthrough in high-precision interferometric measurement technology. In recent years, new-generation SAR constellations such as the PIESAT constellation, Siwei Gaojing constellation, Taijing constellation, and Tianyi constellation have been successively established, featuring high revisit rates and high-resolution imaging capabilities, further expanding the practical applications of InSAR in disaster monitoring, urban management, and national land surveying. Looking forward, China is actively planning new systems, including P-band SAR, dual-antenna InSAR, geosynchronous orbit satellites, and High-Resolution Wide-Swath (HRWS) satellites, continuously promoting the enhancement of constellation deployment and multi-frequency collaborative observation capabilities.

This article examines emerging trends in interferometric synthetic aperture radar technology and systems. It mainly starts with repeat-pass interferometry and single-pass interferometry and goes on to explore new technologies of synthetic aperture radar interferometric measurement.

## 2. InSAR Principle

SAR uses range-oriented large-bandwidth pulse compression technology and azimuth-oriented aperture synthesis technology to acquire ground object echoes. After image processing, it can realize the observation and characteristic identification of a ground image with high resolution. The SAR image signal can be expressed as

(1) $$s=K\exp\left(-j2\pi \frac{r_{Tx}+r_{Rx}}{\lambda }\right),$$

In the formula, $r_{Tx}$ and $r_{Rx}$ are the distances from the phase center of the transmitting radar antenna and the receiving radar antenna to the target; λ is the radar wavelength; and K is a complex number related to the backscattering of the target and the radar equation. For bistatic radar, $r_{Tx}=r_{Rx}=r$. InSAR achieves high-precision relative ranging through the interferometric processing of two or more pairs of interferometric SAR images obtained in the same area. Taking two pairs of bistatic SAR images as an example, after interferometric processing, the main and auxiliary SAR images can be expressed as

(2) $$I_{F}=s_{1}\cdot s_{2}^{*},$$

where $s_{1}$ and $s_{2}$ are the master SAR image and slave SAR image, respectively.

Then, the interference phase can be expressed as

(3) $$\varphi =angle\left(I_{F}\right)=4\pi \frac{r_{2}-r_{1}}{\lambda },$$

where $angle\left(•\right)$ is the phase angle operator; $r_{1}$ and $r_{2}$ are the oblique distance between the phase center of the master and slave SAR antennas and the target, respectively.

From Equation (3), it follows that

(4) $$r_{2}-r_{1}=\frac{\varphi }{4\pi }\lambda ,$$

The accuracy of interferometric phase measurement is related to the phase correlation number *γ* of the master SAR image and the slave SAR image, and

(5) $$\gamma =\frac{\left|E\left(s_{1}\cdot s_{2}^{*}\right)\right|}{\sqrt{E\left({\left|s_{1}\right|}^{2}\right)E\left({\left|s_{2}\right|}^{2}\right)}},$$

where *E* (⋅) is the expected function.

The interference phase probability density function of the master SAR image and the slave SAR image is

(6) $$p(\varphi )=\frac{\Gamma \left(N+\frac{1}{2}\right){\left(1-\gamma^{2}\right)}^{N}\gamma \cos\varphi }{2\sqrt{\pi }\Gamma \left(N\right){\left(1-\gamma^{2}\cos^{2}\varphi \right)}^{N+\frac{1}{2}}}+\frac{{\left(1-\gamma^{2}\right)}^{N}}{2\pi }f\left(N,1;\frac{1}{2};\gamma^{2}\cos^{2}\varphi \right),$$

where N is the multi-look number; Γ (⋅) is the gamma function; and *f* (⋅) is a Gaussian hypergeometric distribution function.

Finally, the standard deviation of the interferometric phase can be expressed as

(7) $$\sigma =\sqrt{\int_{-\pi }^{\pi }\varphi^{2}p(\varphi )d\varphi },$$

## 3. Status and Application

The technique of interferometric synthetic aperture radar is to find the surface change by comparing two radar images acquired at different times or positions. It is mainly divided into repeat-pass interference technology and single-pass interference technology, which provide critical observational capabilities for natural disaster monitoring, glacial dynamics assessment, hydrological resource management, land use/cover change detection, etc., and also provides data support for the study of climate models, marine coral reef degradation, and other fields.

### 3.1. Repeat-Pass Interference

**Figure 2:**
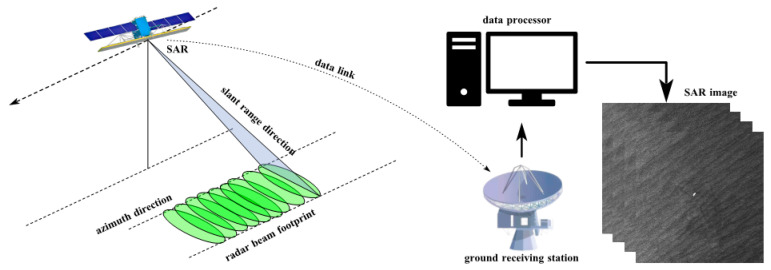
Spaceborne SAR system.

A spaceborne SAR system generally uses a single antenna to collect signals. As shown in Figure 2, for a local area, only one SAR image can be obtained by one satellite flight. Satellite radar repeatedly images the area with a certain time interval and slight orbital deviation, and SAR images obtained twice can form an interference pair. This configuration is called a single-antenna repeating orbit interference system. After acquiring the initial data of the spaceborne radar system, SAR images can be formed only after computer focusing and filtering [20]. With the in-depth study of InSAR technology, the concept and idea of Differential Interferometric SAR (D-InSAR) was born.

#### 3.1.1. D-InSAR

In 1989, Gabriel et al. [21] first proposed the theory of combining interferometry with differential methods to measure surface deformation. They successfully applied this technique to obtain surface deformation data from an irrigation area in California, demonstrating the capability of D-InSAR for high-precision surface deformation monitoring. In 1993, Massonnet and colleagues [22] conducted a study on the Landers earthquake in California, where they used D-InSAR for the first time to extract co-seismic deformation fields, marking the beginning of InSAR’s application in the geosciences. Their results were published in *Nature*, highlighting InSAR’s potential and leading to its widespread use in surface deformation monitoring. However, InSAR is highly sensitive to factors such as Atmospheric Phase Screen (APS) and temporal/spatial decorrelation. To overcome these limitations and enable the high-precision, long-term monitoring of surface deformations, researchers began focusing on these challenges after 2001. These efforts ultimately led to the development of the various Multi-Temporal InSAR (MT-InSAR) techniques.

The data processing procedure for extracting InSAR deformation information generally includes steps such as the registration of primary and secondary SAR images, generation and filtering of the interferogram, removal of the flat-earth phase, topographic phase removal (i.e., differential processing), phase unwrapping, geometric transformation, and geocoding [23,24]. The following will focus on the application of D-InSAR in monitoring and analyzing surface deformation caused by seismic activities, volcanic movements, glacier drift, ground subsidence, landslides, etc. For the description and classification of different types of surface deformation, Table 1 presents typical geophysical phenomena and their corresponding surface deformation characteristics. It can be seen that the deformation characteristics presented by different geophysical phenomena are quite different. Before implementing D-InSAR surface deformation detection, investigating and understanding the temporal and spatial characteristics of the monitoring object can guide the selection of appropriate SAR data and the setting of appropriate processing parameters in the data processing process, and help with the correct interpretation of the deformation field.

In fact, during the SAR interferometric modeling process, the SAR interferometric phase can be regarded as the comprehensive contribution of three factors: the reference ellipsoid, topographic undulation, and surface deformation. On the one hand, this is because the low-frequency high-correlation feature of the atmospheric delay phase in spatial distribution can be eliminated by high-pass filtering in a limited research area [4], while the noise phase can be attributed to systematic errors such as sensor thermal noise and can be suppressed by low-pass filtering, densified ephemerides, etc. [25]. Therefore, D-InSAR to measure ground surface deformation is used to model the interferometric phase of SAR images and sequentially remove the flat-earth phase and topographic phase. Of these, the flat-earth phase can be calculated and removed based on the SAR satellite orbit parameters and imaging geometric parameters, and the topographic phase can be modeled and removed based on external DEM data or the topographic data generated by another SAR interferometric pair, according to SAR imaging parameters.

**Figure 3:**
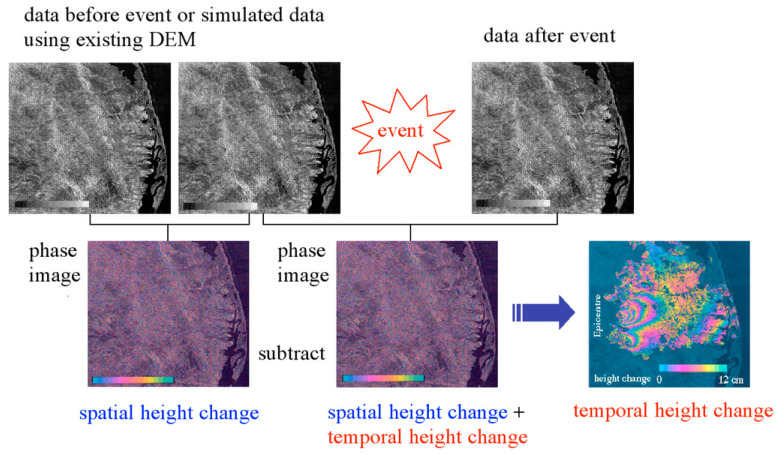
Flow of the measurement of surface elevation change by D-InSAR [4].

Generating differential interferograms is the primary goal of D-InSAR for monitoring ground surface deformation. Figure 3 shows the basic strategy for generating differential interferograms, corresponding to the co-seismic deformation field of the 1995 Sakhalin Island earthquake in Russia [4], which is composed of interferograms formed by JERS-1 SAR data. With the phase information of the master SAR image and slave SAR image, and with the assistance of DEM data and orbit data, through differential interferometry processing, differential interferograms reflecting ground surface deformation can be obtained. It can be seen from this that the phase of the differential interferograms shows periodic fringe changes, and each cycle represents a fringe, indicating a phase change of 2π radians, corresponding to half a radar wavelength of deformation.

**Figure 4:**
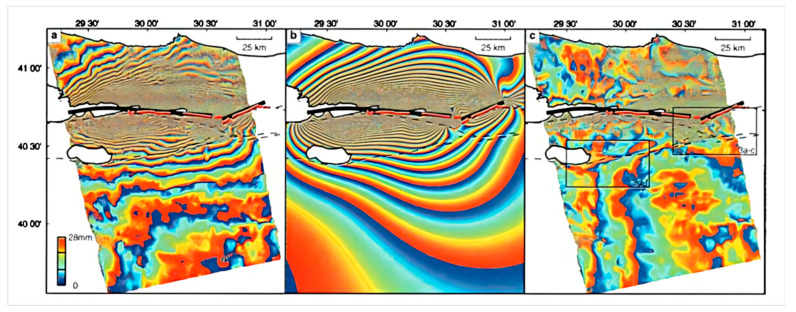
The 1999 Türkiye Mw7.1 Izmit earthquake by D-InSAR [28].(**a**) Interferogram for the Izmit earthquake in the 35-day period between the two image acquisitions; (**b**) Interferogram calculated using the elastic dislocation model; (**c**) Residual interferogram, obtained after subtracting (**b**) from (**a**).

The size of deformation corresponding to each radian of phase change in the differential interferogram is also called the interferometric sensitivity to displacement of the SAR system. The interferometric sensitivity of the SAR system to deformation is an important indicator for surface deformation monitoring. Taking C-band ERS SAR data as an example [26], a deformation of 1 cm along the radar line corresponds to a radar differential interferometric phase change of 2.2 radians (129°), while the same deformation in the L-band PALSAR differential interferogram corresponds to a differential phase change of 0.5 radians (31°) [27]. It can be seen that the D-InSAR interferometric phase shows high sensitivity to ground surface displacement, with short-wave data exhibiting greater sensitivity than long-wave data. Therefore, if different band SAR data are used to measure the same deformation field, the shorter the radar wavelength, the more fringe periods there will be corresponding to the differential interferogram. Similarly, in the same differential interferogram, the place with a larger deformation gradient will correspond to a denser deformation phase fringe. From the above analysis, by observing the density of phase fringes in the differential interferogram, the concentrated range and intensity of ground surface deformation can be evaluated. Figure 4 shows the differential interferogram of the Izmit earthquake, and the red line segment indicates the location of the fault rupture. Notably, the differential interferogram fringes run through the entire interferogram, and deformation occurred within 100 km around the fault rupture due to the earthquake. Moreover, the fringes near the fault are relatively dense, while the ground surface deformation fringes far from the fault are relatively sparse, which is caused by the larger deformation gradient near the fault compared to the far-field region. According to the rule that a surface deformation of 2.8 cm in the line of sight corresponds to a striped pattern cycle, the number of fringes in the deformation field can be observed to estimate the deformation amount in the epicentral area. However, the excessive density of fringes in the epicentral area challenges the accurate estimation of the ground surface deformation amount in areas using visual interpretation methods. In D-InSAR data processing, phase-unwrapping technology is adopted to restore the absolute phase, thereby accurately measuring the ground surface deformation in the epicentral area.

**Figure 5:**
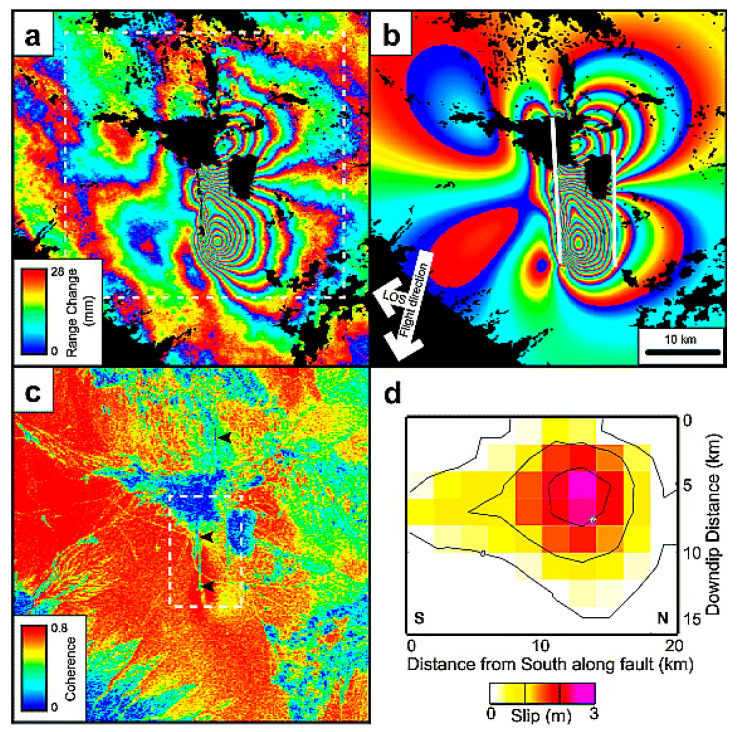
The 2003 Bam Mw6.8 earthquake and deformation by D-InSAR [34]. (**a**) Detail of Envisat ASAR interferogram; (**b**) Interferogram of the same area based upon best-fitting two-fault distributed-slip model; (**c**) Interferometric correlation; (**d**) The distribution of slip on the main right-lateral strike-slip fault.

The most common application of D-InSAR deformation monitoring is in the measurement of co-seismic deformation fields of earthquakes [29]. Since the 1990s, many research institutions have begun to use InSAR to measure the co-seismic deformation fields of earthquakes that have occurred worldwide and have carried out inversion work on sliding fault models [30,31,32]. Pritchard and Simons [33] showed the mosaic map of the co-seismic deformation fields of the four 7.7–8.4 magnitude earthquakes that occurred in Chile from 1992 to 2000 calculated from the ERS-1/2 SAR images, and Figure 5 shows the co-seismic deformation fields and the inversion results of the sliding fault of the 6.8 magnitude earthquake in Bam, Iran, in 2003, calculated from the ENVISAT ASAR images [34]. A large number of studies have shown that the application of InSAR in the monitoring and analysis of co-seismic deformation fields of earthquakes has obvious technical advantages, such as high deformation measurement accuracy and wide coverage. This high-density surface deformation data provides unique key basic data for earthquake mechanism research and model inversion.

It is worth noting that some scholars are exploring the joint monitoring of pre- and post-seismic deformation through combining geodetic techniques such as global navigation satellite system (GNSS) with InSAR technology, attempting to understand the earthquake mechanism more deeply and making new attempts for crustal deformation monitoring and even earthquake prediction [32,35,36]. Compared with conventional geodetic deformation monitoring techniques such as precise leveling and GNSS, InSAR offers characteristics of high accuracy, high resolution, wide coverage, low cost, safety, and continuous observation. It has incomparable superiority to conventional deformation monitoring methods [23,37]. Table 2 lists the comparison between InSAR and precision level measurement and GNSS technologies. Compared with geodetic technologies based on point observations, InSAR provides unique space geodetic measurement technology based on surface observations, which can supplement the existing low-spatial-resolution geodetic measurement technology. Therefore, InSAR provides an economic and effective space Earth observation approach for geophysical research and deformation disaster monitoring.

**Figure 6:**
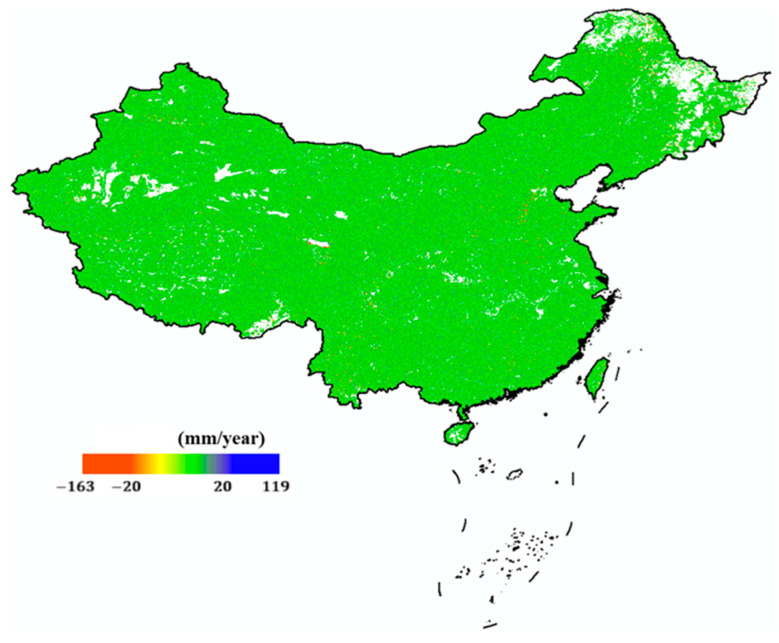
InSAR national deformation map by Wuhan University [38].

China is also providing outstanding results in the field of D-InSAR applications, with a number of research teams producing a series of representative research outcomes. Figure 6 shows in May 2022, Wuhan University released “a map of ground surface deformation across the country with a resolution of 40 m and an accuracy of 5 mm/year” [38], which was produced using Sentinel-1A/B time-series InSAR data covering China in 2021. This achievement was verified by Qianxun Position Network Co., Ltd. through the distribution of more than 2000 base stations of the Beidou ground-based augmentation system in the terrestrial area. The accuracy of the ground surface deformation rate in the Chinese region was 4.82 mm/year. In December 2020, the Aerospace Information Research Institute of the Chinese Academy of Sciences released a national ground subsidy monitoring map based on a supercomputing InSAR system [39,40]. This map is the world’s first of its kind, with extensive coverage and a large amount of processed data. In 2022, Li et al. [41] used InSAR time-series technology to systematically detect earthquake-accelerated landslides triggered by the 2016–2017 Central Italy earthquake sequence. This marked the first large-scale detection of earthquake-accelerated landslides, revealing their long-term response to seismic effects.

#### 3.1.2. PS-InSAR

In 2001, Ferretti et al. [42] introduced the Permanent Scatterers InSAR (PS-InSAR) technique. Using statistical analysis of time-series SAR data over the same area, high-coherence points (i.e., PS points) were detected. By modeling and analyzing discrete PS points, the influence of atmospheric delay could be weakened, and surface deformation and elevation information could be accurately separated. This technique extracts stable and high-Signal-to-Noise-Ratio (SNR) features to eliminate noise and other interferences in the interference map. This method enabled the creation of sub-meter precision surface elevation models and millimeter-level surface deformation monitoring. It has proven especially effective for monitoring infrastructure such as buildings, roads, and dams, often achieving an observation precision of 1 mm or even better [43].

**Figure 7:**
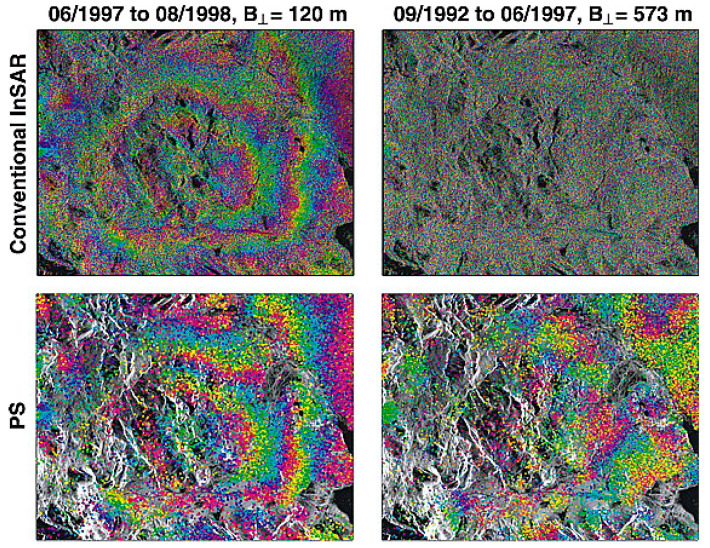
Comparison of wrapped multi-looked interferograms from (**top**) conventional InSAR and (**bottom**) the wrapped phase of individual PS, corrected for DEM error [46].

Ferretti et al. [42,44] successively proposed three methods for identifying permanent scatterers, namely the coherence coefficient threshold method, the amplitude deviation index threshold method, and the phase deviation threshold method. Adam et al. [45] proposed the method using Signal-to-Clutter Ratio (SCR). Hooper et al. [46] proposed the StaMPS method, which selects permanent scatterers by using the maximum likelihood method and compares the results with those of traditional InSAR processing, as shown in Figure 7. Ferretti et al. [47] proposed the SqueeSAR method, which combines PS points and Distributed Scatterers (DS) and solves the problem by using the PS-InSAR processing flow, thereby improving the point density of deformation monitoring and expanding the application field of PS-InSAR technology. These methods are based on large data volumes and select permanent scatterers from a statistical perspective, which are not suitable for data processing with small datasets. Therefore, they limit the application of time series radar interferometry [48,49,50].

#### 3.1.3. SBAS-InSAR

**Figure 8:**
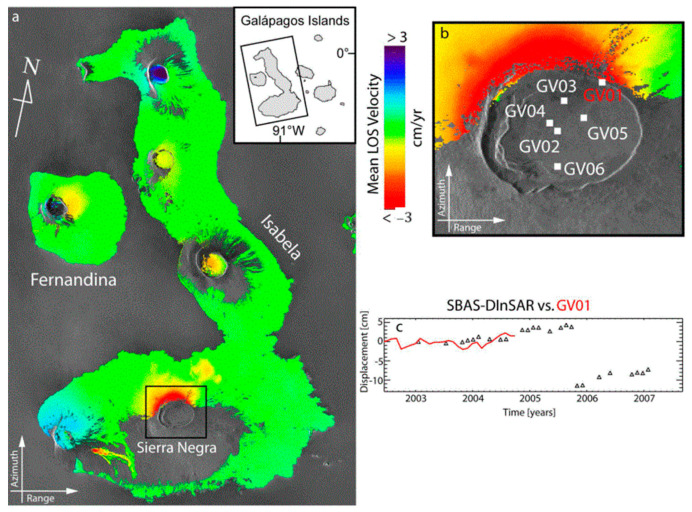
SBAS-InSAR results. (**a**) Mean deformation velocity map. (**b**) Zoom-in of Sierra Negra caldera. The white marks indicate the locations of the stations in the continuous GPS network that has been deployed since 2002. (**c**) Comparison between the SBAS-InSAR time-series (black triangles) and the LOS projected GPS measurements (red line) in correspondence with the GPS station labeled as GV01 [its location is shown in (**b**)] [53].

In 2002, Berardino et al. [51] proposed the Small Baseline Subset (SBAS) method. By selecting short time–space baseline interferograms, the SBAS method limited the decoherence problem caused by long time–space baselines [52]. Compared with PS-InSAR technology, the SBAS method selects more interferograms for calculation. Based on the interferogram phase of the high coherence points in the time series, a deformation model is established. As shown in Figure 8 [53], the overall average deformation velocity map during the study period was obtained through SBAS-InSAR analysis. This map reveals that the D-InSAR technology can only present the local information of the deformation condition of the Sierra Negra crater. In fact, from Figure 8c, the terrain change situation on the north side of the volcano can be clearly observed, and the displacement is approximately a few centimeters. Due to the existence of correlated observations in the interferogram phase, the Singular Value Decomposition (SVD) method is adopted to solve the deformation information and elevation correction amount. This method requires multi-view processing of SAR data, so it is mostly used in large-scale surface subsidence surveys [54,55,56,57,58,59,60].

In conclusion, D-InSAR, SBAS-InSAR, and PS-InSAR are all used to obtain surface deformation information, but there are differences in application range, accuracy, and data processing. Table 3 shows the details.

### 3.2. Single-Pass Interference

**Figure 9:**
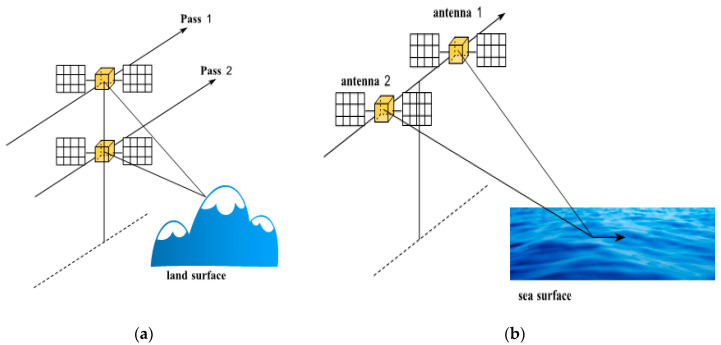
Geometry of repeat-pass. (**a**) CT (Cross-Track)-InSAR; (**b**) AT (Along-Track)-InSAR.

The single-pass mode enables the simultaneous acquisition of dual-channel SAR data in a single flight by installing two antennas on the same platform. It has the advantages of being unaffected by time decoherence, having less atmospheric interference, and having a stable baseline. The single-pass mode can be further classified into Cross-Track Interferometry (XTI) and Along-Track Interferometry (ATI) based on the different baseline structures. XTI refers to the working mode where the baseline is perpendicular to the heading direction, as shown in Figure 9a, and in this mode, one antenna transmits electromagnetic waves while the other two antennas simultaneously receive the reflected information from the ground. The interference phase is caused by the path difference between the two antennas and the ground target, and this path difference has a geometric relationship with the terrain. Therefore, this mode is used to obtain elevation information of the ground surface. ATI mode refers to the working mode where the baseline is parallel to the heading direction, as shown in Figure 9b, and the interference phase is mainly caused by the displacement changes of the ground target within two observations. Thus, this mode is often used for detecting ground target movement, mapping sea surface currents, etc. It is difficult to build two SAR systems with a certain baseline distance on the spacecraft platform, so this mode is mainly used on airborne platforms.

#### 3.2.1. CT-InSAR

Compared with optical satellites, InSAR systems basically do not have limitations on imaging ground targets due to conditions like day–night cycles and weather. Especially in areas with perennial rain, uninhabited areas, and foreign regions, InSAR systems have unparalleled advantages. Compared with SAR stereo mapping technology, the InSAR technology with phase measurement has higher measurement accuracy. Table 4 lists the comparison between InSAR and other DEM acquisition technologies. Terrain measurement using InSAR technology can already achieve operational applications. Examples are the SRTM system, TanDEM-X system and the mapping project for the blank areas of 1:50,000 topographic maps in western China, etc.

**Figure 10:**
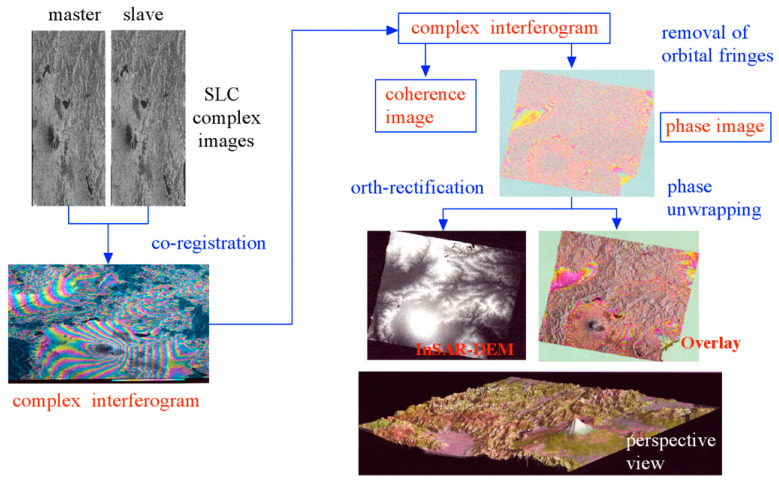
Flow of CT-InSAR DEM generation [4].

Since interferometric measurement requires two SAR images with high coherence in the same region, and the interferometric phase cannot be directly obtained from the complex images, the generation of digital surface models (DSM) requires several steps such as complex image registration, interferogram generation, interferometric phase filtering, phase unwrapping, baseline estimation, and DSM reconstruction. The specific processing flow is shown in Figure 10.

In 2000, the SRTM system of the United States [61,62] acquired SAR data ranging from 60° N to 56° S. This system extended a 60 m telescopic boom to project an X-band and a C-band antenna outside the cabin, forming a dual-antenna InSAR system with the main antenna inside the cabin. Using InSAR technology, it produced DEM products covering 80% of the global land surface. The product had a planar resolution of 30 m × 30 m, a relative elevation accuracy of 6 m, and an absolute elevation accuracy of 16 m, meeting the DTED-2 standard in Table 5.

In 2010, the German satellite TanDEM-X was successfully launched and operated in a HELIX configuration with the previous TerraSAR-X satellite [63,64,65], pioneering true spaceborne dual-station SAR interferometric measurement. Compared with SRTM data, the TanDEM-X DEM expanded from 60° N to 56° S to cover the entire globe, including all regions of the North and South Poles, and the spatial resolution and elevation accuracy increased to 12 m and 2 m, respectively, meeting the HRTI-3 standard in Table 5.

China has also actively conducted research and application explorations in this field, achieving groundbreaking progress. The TianHui-2 dual-satellite formation launched in 2019 [66,67] has a payload of high-resolution synthetic aperture radar, high-precision inter-satellite relative state measurement equipment, etc. It acquires global radar images and auxiliary measurement data in all-weather and all-time conditions, enabling the rapid production of DSM.

**Figure 11:**
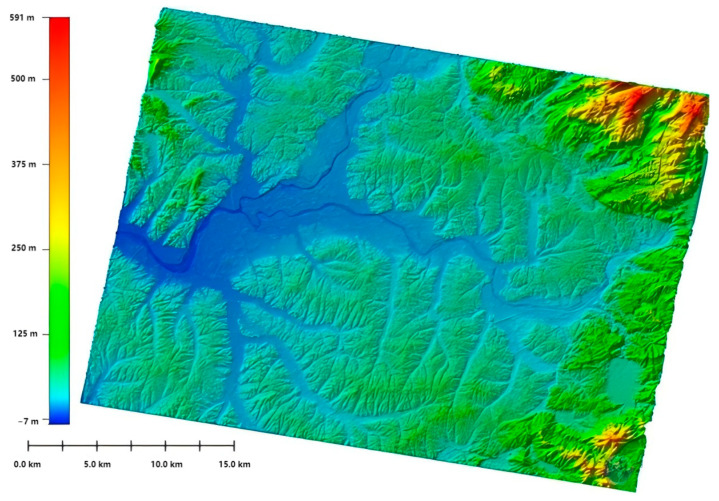
Yueyang County, Hunan Province, DSM generation [68].

The PIESAT-1(01) satellite launched in 2022 was developed by Galaxy Aerospace. This satellite system adopted the world’s first “1 + 3” wheel formation multi-satellite distributed interferometric configuration, consisting of 4 high-resolution X-band radar satellites, and has the capabilities of high-precision terrain mapping, high-resolution wide-angle imaging, high-precision deformation monitoring, etc. It can quickly and efficiently produce high-precision DSM and complete global non-polar region mapping tasks, and is widely applied for domestic emergency disaster reduction, ecological environment, natural resources and other scenarios. Through the elevation accuracy verification of the RAW DSM products in Yuyang County, Hunan Province, using ICESAT2 laser control point data, the absolute elevation mean error is found to be 3.34 m, meeting the terrain mapping accuracy requirements of 1:50,000 scale in hilly areas. The RAW DSM products in Yuyang County, Hunan Province, are shown in Figure 11.

#### 3.2.2. AT-InSAR

The Along-Track Interferometry method involves placing two antenna pairs along the flight path to image the same scene. Each antenna processes its own image. Due to the ocean surface movement, there will be a phase difference between the two images. This phase difference can be used to measure the line-of-sight velocity of ocean surface currents, as shown in Figure 9b. In 2000, Romeiser discussed the feasibility and limitations of ATI flow measurement through model simulation [69], and in 2010, he applied AT-InSAR to TerraSAR-X. The data processing results verified the effectiveness of the along-track interferometry method in current field measurement and inversion [70].

In February 2000, the SRTM, which was mainly used for the precise mapping of global terrain, began its mission. The aircraft placed the main antenna inside the cabin and the secondary antenna outside, forming a 60 m long cross-track interferometry baseline and a 7 m long in-track interferometry baseline [61]. Romeiser and Thompson conducted numerical simulation studies on the imaging mechanism of AT-InSAR for ocean surface currents, proving the feasibility and limitations of AT-InSAR in measuring the radial velocity of ocean surface currents; that is, the along-track interferometry phase is not simply proportional to the radial velocity of ocean surface currents in the line-of-sight direction. It also includes phase deviations caused by Bragg waves, ocean waves, and sea surface wind fields [69]. Romeiser also proposed a composite ocean surface model for calculating the normalized radar backscattering cross-section (NRCS) of the ocean surface under medium incidence angles. This model incorporates the analysis of sea surface wind fields and Bragg waves [71,72].

Based on this, Romeiser et al. demonstrated the feasibility of airborne AT-InSAR for ocean surface current measurement using SRTM interferometric data from the Waddenzee region in the Netherlands and KUSTWAD current field data [73]. They further demonstrated its application potential with SRTM interferometric data from the Orkney Islands in northern Scotland, launching the DLR’s AT-InSAR mission (TerraSAR-X) [74]. They comprehensively discussed the theoretical ocean surface current measurement capabilities of TerraSAR-X AT-InSAR mode, and the results proved that the ocean surface current measurement capability of TerraSAR-X AT-InSAR mode was at least comparable to that of SRTM [75].

At the European Conference on Synthetic Aperture Radar (EUSAR) in 2022, Ahmed et al. presented an analysis report on the Radarsat-2’s MODEX-1 ATI mode and Doppler centroid anomaly (DCA) for estimating the radial velocity of ocean surface currents. The paper processed the Agulhas current data obtained by Radarsat-2 MODEX-1 in 2021, and the processing results are shown in the following figure. Under the condition of approximately equal accuracy, the spatial resolution of ATI is ten times higher than that of DCA [76].

To obtain the two-dimensional ocean surface current, two sets of mutually perpendicular radial velocity components of the ocean surface current need to be simultaneously acquired. In 1994, Rodriguez et al. [77] first mentioned the extended method of ATI-SAR for estimating ocean surface current, and in 2001, Frasier and Camp named it dual-beam interferometer (DBI). It can achieve the acquisition of two-dimensional ocean surface current with a single pass [78].

**Figure 12:**
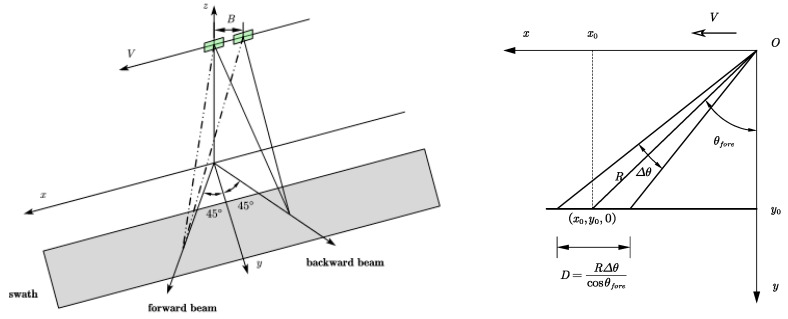
The observation geometry of the DBI method.

The observation geometry of the DBI method is shown in Figure 12. In the coherent radar, a pair of dual-beam antennas are adopted, and each antenna generates a forward and a backward beam. Due to the forward motion of the flight platform, the echo frequency of the forward beam increases, and the echo frequency of the backward beam decreases. The time required for the spatially registered backward antenna echo and forward antenna echo to move between each other is determined according to the traverse antenna baseline distance B. The radar echoes from the two forward beams and the radar echoes from the two backward beams are cross-correlated, generating a pair of interferograms. The phase of each interferogram provides a line-of-sight component of the Doppler surface velocity, and then the vector estimation is obtained by appropriately combining the separated interferograms.

As shown in the right part of Figure 12, the radial velocity measurement of the target point in the forward beam is as follows:

(8) $${\hat{u}}_{r}=\frac{V}{kB}\phi ,$$

Then,

(9) $$x_{\max}=x_{0}+\frac{R}{V}u_{r},$$

Then, the phase at the point $x_{\max}$ is

(10) $$\phi (x_{\max})=\frac{kB}{V}u_{r}=k\tau u_{r},$$

Herein, k represents the wave number, τ represents the coherence time, and τ=B/V. From this, the radial velocity measurement formula can be derived. ${\hat{u}}_{r}$ represents the estimated value of radial velocity. According to the geometric relationship, the radial velocities measured by the forward and backward beams can be used to calculate the two-dimensional ocean surface currents, which are the velocity components of the ocean surface currents along the track and perpendicular to the track, respectively:

(11) $${\hat{v}}_{Ox}=\frac{{\hat{u}}_{r}^{+}}{2\sin\theta_{i}\sin\theta_{fore}}-\frac{{\hat{u}}_{r}^{-}}{2\sin\theta_{i}\sin\theta_{aft}},{\hat{v}}_{Oy}=\frac{{\hat{u}}_{r}^{+}}{2\sin\theta_{i}\cos\theta_{fore}}+\frac{{\hat{u}}_{r}^{-}}{2\sin\theta_{i}\cos\theta_{aft}}$$

Among them, $\theta_{fore}=\theta_{aft}=\theta_{ps}$ is called the projection squint angle. The variance of radial velocity can be obtained by measuring the radial velocity from the forward and backward wave beams:

(12) $$\sigma_{v_{ox}}^{2}=\frac{\sigma_{u_{r}^{+}}^{2}+\sigma_{u_{r}^{-}}^{2}}{{\left(2\sin\theta_{i}\sin\theta_{ps}\right)}^{2}},\sigma_{v_{oy}}^{2}=\frac{\sigma_{u_{r}^{+}}^{2}+\sigma_{u_{r}^{-}}^{2}}{{\left(2\sin\theta_{i}\cos\theta_{ps}\right)}^{2}},$$

The derivation of the phase yields the overall variance of the velocity as follows:

(13) $$\sigma_{v}^{2}=\frac{\sigma_{\phi^{+}}^{2}+\sigma_{\phi^{-}}^{2}}{{\left(k\tau \sin\theta_{i}\sin2\theta_{ps}\right)}^{2}}$$

At the International Geoscience and Remote Sensing Symposium (IGARSS) in 2011, Christopher Buck introduced the Wavemill project. In November 2011, an airborne test was conducted to verify the feasibility of the squint InSAR method. Further potential was explored to optimize the system design [79]. Regarding the airborne test of the Wavemill project, Yague-Martinez et al. [80] presented the system parameters and processing algorithms of the airborne test at the EUSAR conference in 2012. The airborne test area was in Liverpool Bay and Anglesey, UK.

The formation along track interference has the characteristics of flexible multi-baseline, mixed baseline, and multi-angle observation. It can measure ocean surface currents, sea surface wind fields, and ocean waves while also conducting elevation mapping of the sea surface.

In June 2010, TanDEM-X (TDX) was launched into space. Together with TerraSAR-X (TSX), which was launched earlier, they formed a dual-star formation in a helix configuration. The main task of the TSX and TDX radar system formation was to map the global DEM. The working distance could be controlled within 150 to 400 m [63,64,81,82]. In addition, the TSX and TDX radar system formation could provide a baseline along the flight path within a range of zero to a few kilometers, because TDX also had a dual-reception mode. After forming a dual-star formation with TSX, it could be equivalent to having four phase centers along the flight path for interferometric SAR with long and short baselines. The design of long and short baselines can effectively solve the phase ambiguity problem of high-speed scatterers [13]. The long baseline is sensitive to slow motion while the short baseline is sensitive to fast motion.

In early 2012, Romeiser and Suchandt [83,84] obtained two sets of data with nearly optimal baselines from the TDX satellite formation. The imaging area was in the northern part of Scotland and the Orkney Islands to its north. In addition to the along-track interferometric data from the TDX satellite formation, Aperture Switching (AS) mode data from the TSX satellite was acquired in this area in 2009, and DRA mode data from the TSX satellite was acquired in this area in 2010. By comparing the processing results of TSX satellite DRA mode data processed by DCA and TDX satellite formation data processed by ATI, it was found that the DRA mode must be averaged within a resolution of 1000 m × 1000 m to achieve an error of 0.1 m/s, while in the ATI mode, an error of 0.1 m/s can reach a resolution of 33 m × 33 m. The results show that the accuracy of the ocean surface current of DCA is lower than that of the ATI method, approaching the accuracy of the short-baseline ATI method. DCA is an effective alternative method to the single-star dual-channel receiving antenna mode ATI. The ATI processing results of the TDX satellite formation prove the true potential of ATI technology when approaching the optimal baseline [85].

**Figure 13:**
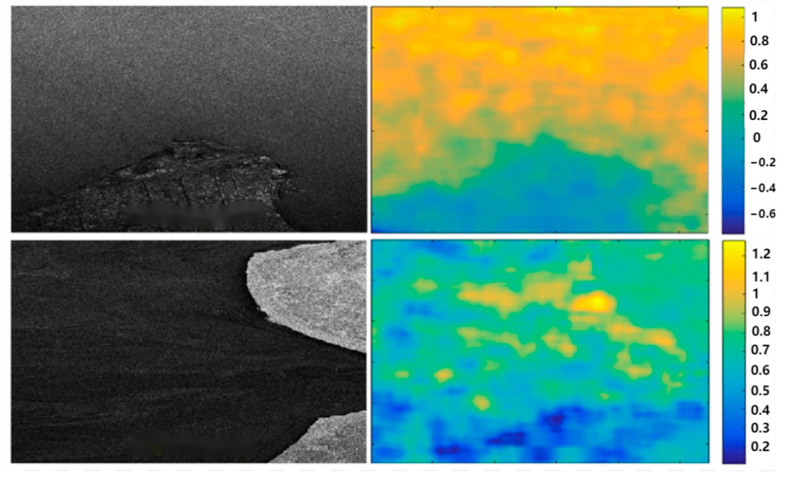
The measurement results of ocean surface currents by the ATI mode of the GF-3 satellite.

**Figure 14:**
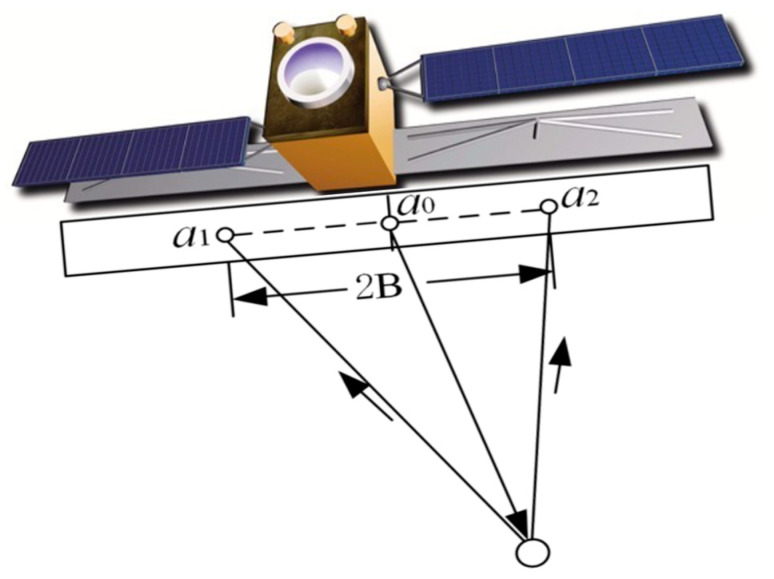
Schematic diagram of GF-3 ATI mode.

China has also made significant breakthroughs in the field of AT-InSAR. The GF-3 satellite demonstrates excellent capability for AT-InSAR. The satellite was equipped with a 15 m × 1.232 m four-polarization waveguide slot phased array SAR antenna [86]. The antenna array surface was divided into four deployable panels, and each panel contained six identical modules, and each module contained sixteen unit dual-polarization waveguide slot subarrays, supporting the single-antenna dual-aperture ATI experimental mode [87]. In 2019, Yuan Xinzhe et al. [88] conducted the first experiment measuring ocean surface currents using the ATI mode of a GF-3 satellite in Jiaozhou Bay, China, see Figure 13. As shown in Figure 14, the full-aperture transmitting radar signal was emitted, and the echoes were received simultaneously by two sub-apertures divided in half along the orbit. The effective baseline of the interferometric measurement was 3.75 m. The ECMWF (European Centre for Medium Weather Forecasts) wind field was used for the on-site wind speed, and the root mean square error between the HFSWR (High Frequency Surface Wave Radar) data and the measured values was less than 0.2 m/s [88].

## 4. Trends and Challenges

### 4.1. Multidimensional

#### 4.1.1. Multi-Satellite Networking

Since the launch of the first satellite, Sentinel-1A, in April 2014, the Sentinel-1 series has played a significant role in the field of Earth observation. In 2016, the second satellite, Sentinel-1B, was successfully launched, further enhancing the mission’s capabilities. However, Sentinel-1B ended its mission early in August 2022 due to a technical glitch that prevented it from continuing to acquire data. The third Sentinel-1 satellite (Sentinel-1C) was successfully launched on 5 December 2024 by a Vega-C rocket from the European Spaceport in French Guiana. Equipped with the most advanced C-band SAR instrument, Sentinel-1C is capable of providing high-resolution images of the Earth’s surface at all times and in all weather conditions, supporting a variety of critical applications such as environmental management, disaster response, and climate change research. In addition, Sentinel-1C introduces an automatic identification system (AIS) to enhance maritime traffic detection and monitoring capabilities, enabling accurate tracking of a ship’s identity, location, and direction of travel. A dual satellite constellation (consisting of two identical satellites in the same orbit but 180° apart) can be reformed to ensure the continuity and efficiency of Earth observation data [89].

**Figure 15:**
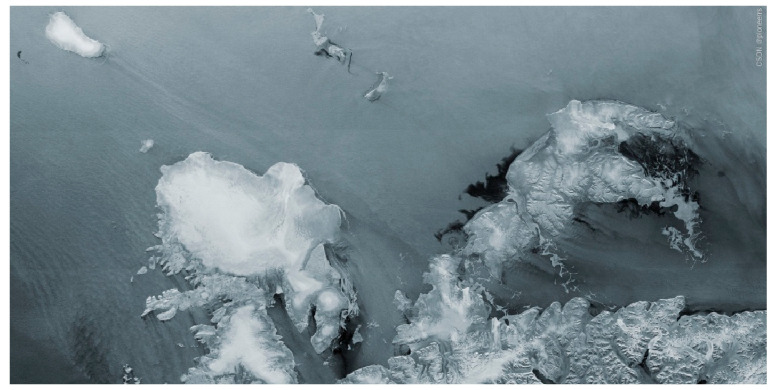
Norway’s Svalbard islands are covered by ice caps [image credit: ESA].

A large number of studies have shown that Sentinel-1 can continuously and effectively monitor surface deformation. Figure 15 shows Sentinel-1C’s ability to monitor ice cover and environmental changes in harsh remote areas [90].

In terms of China, we have progressed greatly in the constellation development of SAR satellites. The first notable example is GF-3. Following the successful launch and operation of GF-3 in 2016, GF-3B and GF-3C were launched in November 2021 and April 2022, respectively, marking the establishment of China’s first SAR satellite constellation. Both GF-3B and GF-3C completed their in-orbit commissioning in February 2023 and entered the operational phase. The new satellites introduced several system upgrades, including the integration of an Automatic Identification System (AIS) receiver for ship tracking, onboard real-time data processing, additional imaging modes, an increased maximum duty cycle, and enhanced support for maritime monitoring and InSAR applications. The coordinated operation of the three satellites significantly improves data revisit frequency and response time, while also enriching data diversity, thereby providing strong support for applications such as marine environment monitoring, disaster response, and deformation mapping [18].

Existing research has demonstrated that the coordinated operation of the GF-3 constellation exhibits improved interferometric performance. For instance, an interferometric pair between GF-3 and GF-3B with a short revisit interval of six days, despite a large perpendicular baseline, was able to produce a coherent interferogram. This capability highlights the advantages and potential of the GF-3 constellation for high-frequency, all-weather, and long-term SAR observation missions [19].

In addition to GF-3, recently launched missions such as LT-1 and PIESAT have also been developed as constellations. The success of these missions marks the development and advancement of China’s SAR satellite programs.

#### 4.1.2. Multiband

**Figure 16:**
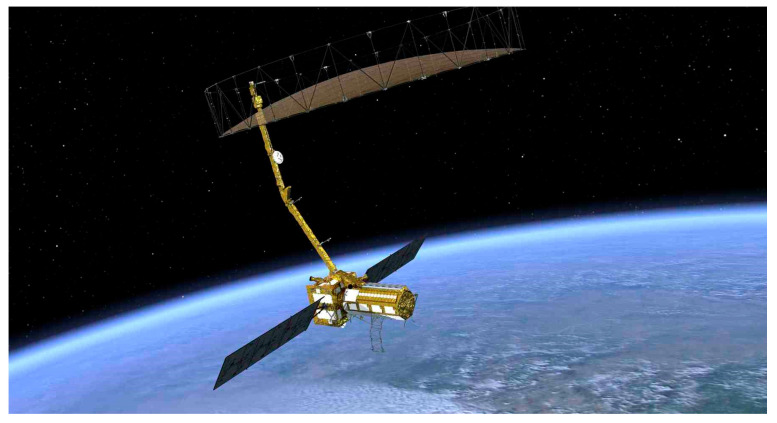
NISAR satellite [image credit: courtesy of NASA/JPL-Caltech].

The NASA-ISRO Synthetic Aperture Radar (NISAR) as shown in Figure 16 is scheduled to be launched in 2025 [91]. Developed by NASA and ISRO, it is primarily designed to monitor almost all land and ice surfaces on Earth every 12 days. Equipped with a dual-frequency radar system (L-band and S-band), it is used to track vegetation changes, measure ice sheet dynamics, monitor natural disasters such as earthquakes and landslides, and study long-term changes in the Earth’s structure and climate [92,93].

China is currently progressing with the testing of multiband SAR systems on airborne platforms and has made significant advancements. Zhou et al. [94] developed a representative airborne multi-dimensional SAR observation platform (MSJosSAR), which integrates X, C, and L-band SAR to enable coordinated multi-band imaging. By combining the scattering characteristics of different bands, the system enhances target recognition, penetration, and scene adaptability. A unified frequency source ensures time and phase synchronization across bands, supporting data fusion and interferometric applications. MSJosSAR also supports full-polarization imaging, variable incidence angle observation, and repeat-pass interferometry. Automatic registration across bands is achieved through error modeling and self-calibration, with sub-pixel accuracy. The platform demonstrates strong performance in multi-dimensional structure reconstruction and coherence variation monitoring, laying a solid technical foundation for future satellite-based SAR systems with multi-band, high-resolution, and high-precision capabilities. Lu Li et al. successfully acquired high-resolution SAR data in the P, L, C, and X bands using a multi-frequency SAR sensor system developed by the Aerospace Information Research Institute, Chinese Academy of Sciences, providing data support for ground target classification and Earth observation applications [95]. Liu Bingce et al. designed a compact X/L dual-band, multi-bandwidth receiver scheme to meet the requirements of multi-frequency, multi-mode receivers for UAV platforms. Compared with traditional UAV-borne radar receivers, this design features multi-mode operation, wide signal bandwidth processing capability, and a high level of integration [96].

#### 4.1.3. Multi-Baseline

On a single spaceborne platform, the resolution capability of InSAR is limited. However, by having multiple spaceborne platforms work in coordination, the spatial and temporal resolution of the images can be significantly enhanced, and the formation of satellite constellations can cover broader and more detailed geographical areas. Moreover, formation interferometry technology can dynamically adjust the number of platforms and sensor layouts based on specific mission requirements, and can carry multiple sensors to achieve multi-band monitoring and meet the monitoring needs of different scenarios, thereby enriching the data.

**Figure 17:**
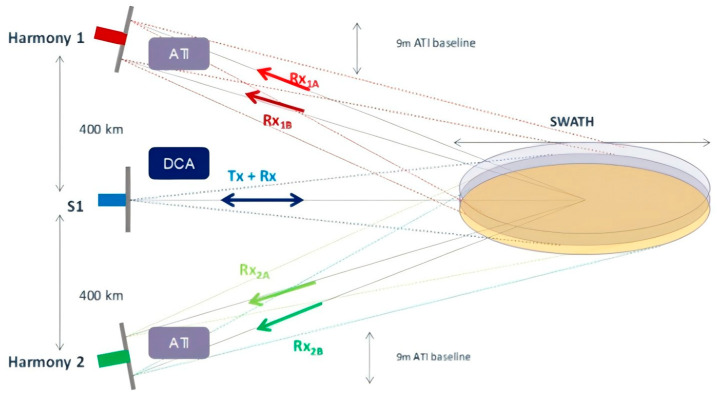
Harmony formation configuration.

In 2019, at the IGRASS conference, the ESA announced Harmony as a candidate for the Earth Observation 10 mission [97,98]. The Harmony project plans to launch two SAR satellites weighing less than 500 kg each, carrying only receivers, to receive echoes from Sentinel-1D. Their orbital configuration will be divided into two phases, as shown in Figure 17. In the first phase, Harmony-A and Harmony-B satellites will form a cross-track baseline and maintain it for over a year to study the seasonal changes in ice layers. In the second phase, B and A satellites will be distributed approximately 250 km ahead and behind Sentinel-1D, forming two pairs of along-track interferometric pairs to achieve a spatial resolution of 1 to 4 km and a sea surface velocity measurement accuracy of 0.15 m/s.

**Figure 18:**
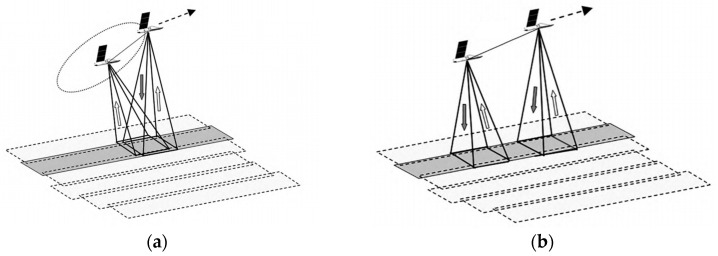
TIANHUI-2 formation configuration. (**a**) Fly-around formation InSAR; (**b**) follow-up formation InSAR.

**Figure 19:**
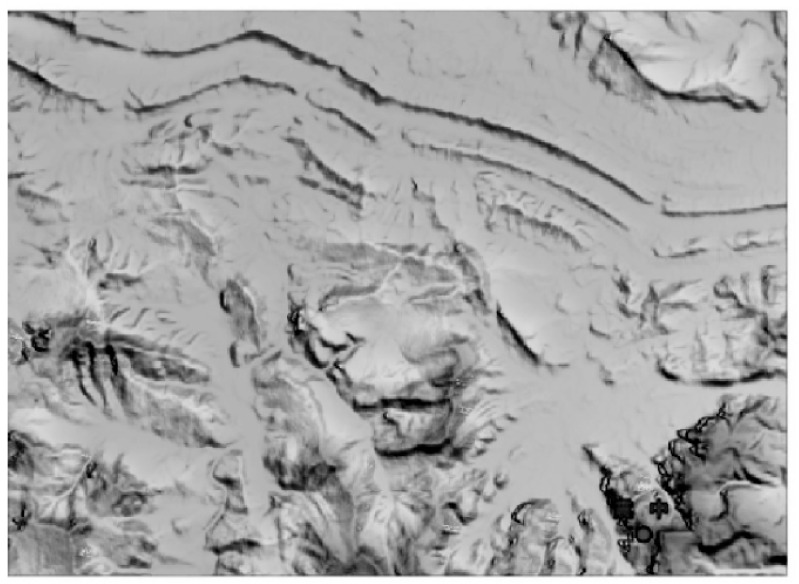
DSM data in follow-up mode.

China has made substantial progress in the development of satellite interferometric observation systems, particularly in formation flying and multi-satellite InSAR technologies. On 30 April 2019, the China TIANHUI-2 satellites were launched. The TIANHUI-2 can obtain InSAR data for the ground area between 75° S and 82° N latitude on the globe, mainly for the production of high-precision DEM, DSM, and radar orthophoto products [66,99]. The InSAR altitude measurement mode of the fly-by formation is the main working mode of TIANHUI-2, as shown in Figure 18. During a single pass, two satellites can obtain the coherent echo data of the same area on the ground in strips, and the data are transmitted to the ground for InSAR altitude measurement processing [100]. In the early stage of orbiting, the two satellites operated in the follow-up mode, with an inter-satellite distance of about 30 km. During this period, the SAR performance indicators of each satellite were tested, and on 17 June, when the Sichuan Yibin earthquake occurred, the distance between the satellites was 5 km, and the satellite follow-up mode images of this area were obtained. DSM data were processed, as shown in Figure 19.

**Figure 20:**
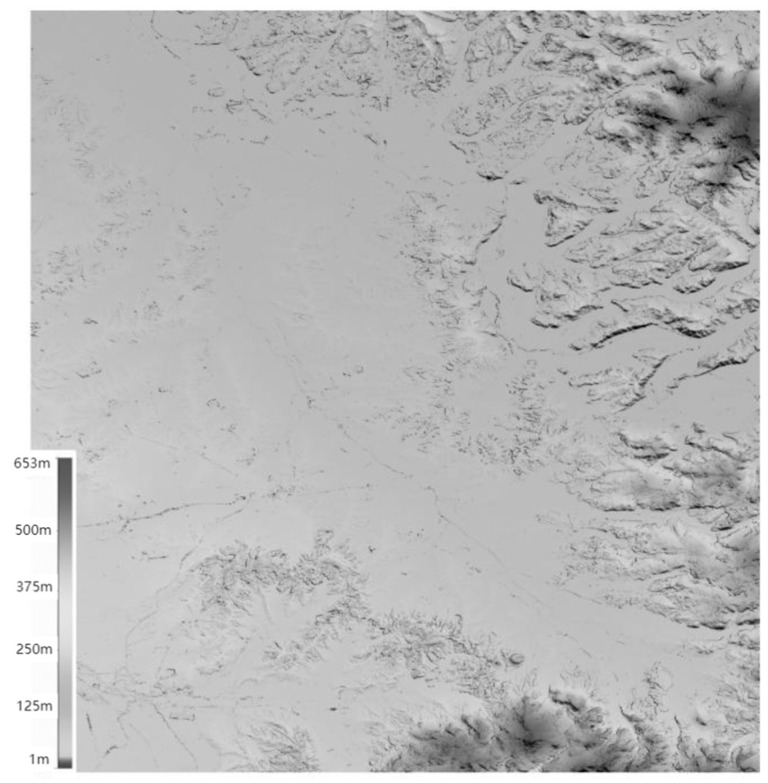
DSM data in fly-around mode.

On 9 July, the system formed a fly-by formation configuration, with an effective baseline of about 500 m. After power-on, InSAR data were obtained that night. The data were transmitted to the ground system, and using self-developed software, the first DSM mapping product was successfully processed at one attempt, as shown in Figure 20. The successful launch of the TIANHUI-2 satellite enabled China’s microwave remote sensing to achieve a stepwise development from single-satellite observation to dual-satellite interferometric mapping, filling the gap in the field of distributed formation radar satellites in China, making China the second country in the world to have a microwave interferometric mapping satellite, and proposing for the first time an international method to solve the absolute ambiguity problem of interferometric phase through a system design of dual-frequency imaging, completely eliminating the dependence on control data. This marked a major breakthrough in the construction of China’s aerospace surveying and mapping capabilities. Its all-weather data acquisition capability, fast data processing speed, etc., allow it to overcome the limitation that optical mapping satellites cannot obtain data in areas with perennial cloudy and rainy weather. Moreover, the rapidly generated DSM data can be directly used for the production of topographic maps and orthophotos of optical mapping satellites. This will greatly accelerate the speed of China’s global geographic spatial information construction [101]. The TIANHUI-2 satellite system adopts a dual-satellite inter-satellite fly-by formation and a one-launch dual-reception interferometric technology system, operating in a Sun-synchronous orbit at an altitude of 500 km. The in-orbit test results show that the system is in good operating condition, and its main performance indicators are superior to the design indicators. Its positioning accuracy is comparable to that of the German TDX system and can be used for the production of 1:50,000 scale geographic spatial information products.

On 30 March 2023, China PIESAT-1 satellite was launched. It is formed of a 1 + 3 wheel interference formation. Its main tasks are global land surveying and mapping, imaging observation, deformation monitoring, and elevation measurement. The elevation measurement accuracy reaches relative elevation accuracy (CE90) ≤ 3 m (flat land, hilly land), 7 m (mountainous land, highland land), and absolute elevation accuracy (CE90) ≤ 7 m. The mapping accuracy is better than 1:50,000, and the deformation accuracy is 3–5 mm/year.

**Figure 21:**
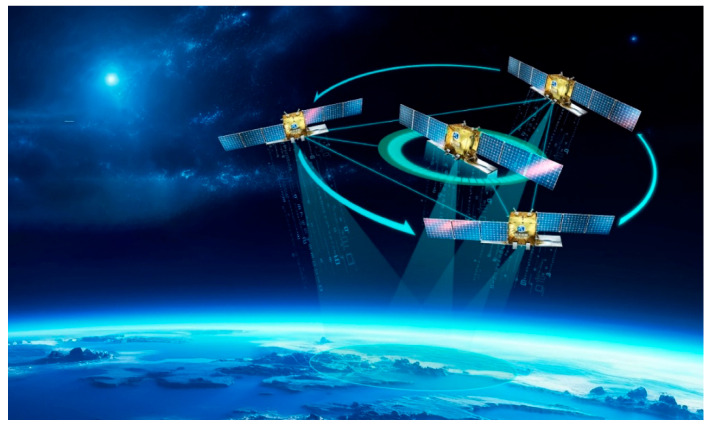
PIESAT-1 satellite formation.

**Figure 22:**
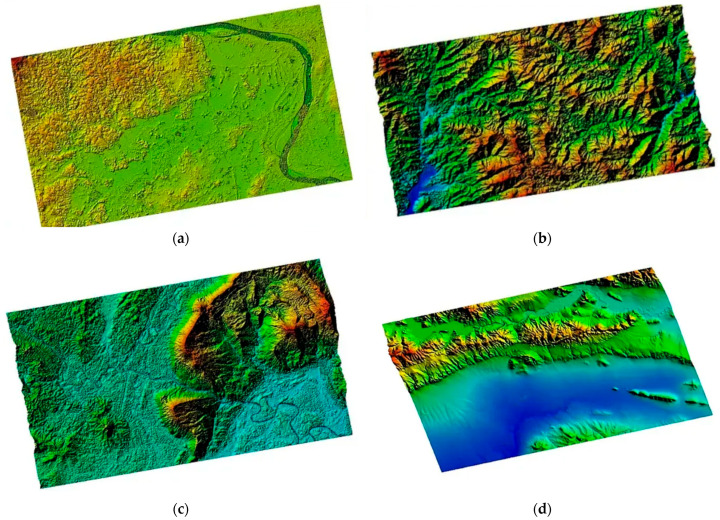
DSM and DEM published by PIESAT Information Technology Co., Ltd. (**a**) Plain area data in Laos; (**b**) hilly area data in Laos; (**c**) mountainous and highland area data in Laos; (**d**) DEM of the central region of Bulgaria.

As shown in Figure 21, PIESAT Information Technology Co., Ltd., Beijing, China, provided the “PIESAT-1” satellite constellation remote sensing image product set in 2024 [102]. On 9 November 2024 and 17 December 2024, four satellites were launched in one rocket in the form of one satellite per group, forming a 90-degree equip phase in-orbit formation, constituting a 1 × 4 Walker constellation system. Among them, one satellite serves as the backup satellite for the “PIESAT-1” main satellite and operates in the main satellite’s orbit, with technical status basically consistent with the main satellite. The PIESAT-2 constellation provides single-satellite high-resolution imaging, multi-satellite high-precision interferometric measurement, on-board data processing and autonomous planning, and emergency communication and rapid response, as well as continuous and stable data production capabilities. It mainly conducts observation tasks such as global regional regular coverage, rapid revisit of specific areas, high-frequency monitoring of key targets, and emergency call for special scenarios, supporting and serving application demands in fields such as land resources, earthquake, disaster prevention and mitigation, acquisition of basic geographic information, and forestry. The satellites have played an important role in multiple emergency events such as monitoring of the rainstorm and flood disasters in the Beijing–Tianjin–Hebei region in 2023, monitoring of the breach of Dongting Lake in Huarong County, Hunan Province, monitoring of rare heavy rain in Huludao, Liaoning Province, monitoring of forest fires and damage in the Abei Forest Farm of the Inner Mongolia Forestry Industry, and monitoring of the collapse of the Meida Expressway in Taubao, Guangdong Province. As shown in Figure 22, they have supported the monitoring of various events more than a hundred times and generated over 500,000 standard image data, covering the entire country, such as Laos.

**Figure 23:**
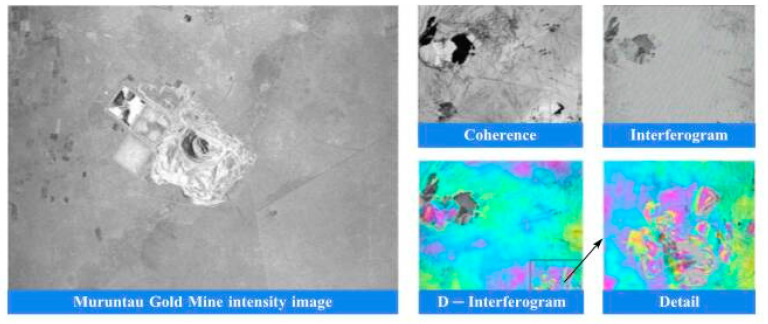
Deformation monitoring of the Muruntau Gold Mine in Uzbekistan.

Based on the DSM products, DEM products are generated. The terrain features of the DEM products are clearly visible. The terrain details of mountains, rivers, and flatlands are very distinct. Moreover, the high-precision DEM products can ensure the accuracy of terrain analysis and the precision of hydrological modeling, providing reliable data support for various industries’ analyses. For example, deformation monitoring of the Muruntau Gold Mine in Uzbekistan plays a crucial role in assessing potential geological disasters and analyzing the mechanism of subsidence in mining areas, see Figure 23.

#### 4.1.4. Multipolarity

In 1997, S.R. Cloude and K.P. Papathanassiou [103,104] first studied the impact of frequency and polarization on coherence using SIR-C/X-SAR data, and found that coherence strongly depends on polarization, which was pioneering research in the field of multipolarization SAR interferometry. Polarimetric SAR interferometry (PolInSAR) offers unique advantages that conventional SAR cannot match, and has gradually become a new research hotspot in the field of SAR data processing [80,105,106,107]. Kugler et al. [106] applied this technology for forest height inversion and validated its effectiveness under different forest structures and seasonal conditions. In recent years, significant progress has been made in the application of PolInSAR technology for surface vegetation height inversion [108]. With the continued use of both full-polarization and dual-polarization synthetic aperture radar data, PolInSAR has expanded its applications in forest biomass estimation, carbon stock assessment, and tropical forest monitoring.

**Figure 24:**
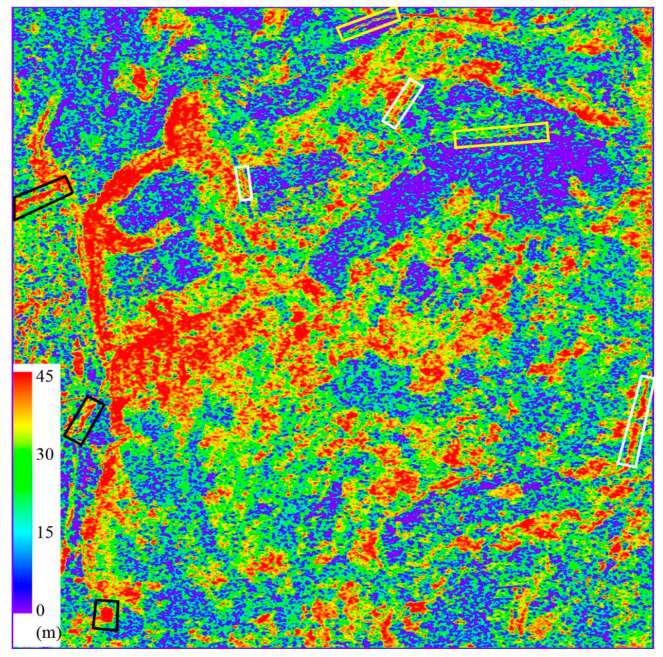
The inversion results of dual polarization SAR data are processed by the interference method [109] (black area is water bodies and yellow area is roads).

**Figure 25:**
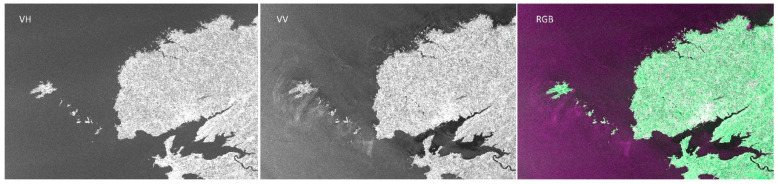
Image acquired by Copernicus Sentinel-1A on 1 November 2017 over the Brittany region, France, with VV intensity image, VH intensity image, and RGB color composite. Copernicus Sentinel data (2017) processed by CLS [89].

In 2015, Fu et al. [109] proposed a PolInSAR technique based on dual-polarization SAR data and successfully applied it to forest height estimation by extending the three-stage inversion process and coherence optimization algorithm. As shown in Figure 24, experimental results show that dual-polarization PolInSAR performs comparably to the full-polarization mode, and the proposed volume-only coherence search method significantly enhances inversion accuracy. Figure 25 shows the grayscale of Sentinel-1A under different polarization modes.

In recent years, China has made significant progress in the development and application of spaceborne PolInSAR systems, gradually establishing a domestic SAR satellite fleet with multi-polarization observation and interferometric measurement capabilities. Studies have shown that the GF-3 satellite’s DInSAR technique can achieve sub-centimeter-level ground deformation monitoring. The integration of polarimetric information further improves the accuracy of surface change detection, showing high consistency with optical monitoring results [110]. Moreover, change detection combining polarimetric data accurately delineates collapse areas in mining regions, with mapped areas closely matching public monitoring data, demonstrating the potential of GF-3′s multi-polarization data in dynamic surface monitoring. Although PolInSAR applications for forestry or agriculture based on GF-3 remain limited, its high-resolution full-polarimetric capability provides a valuable data foundation for future biomass estimation studies.

The LT-1 constellation, as China’s first L-band fully polarimetric PolInSAR system, has achieved remarkable results in polarimetric calibration and interferometric measurement performance. Relevant studies conducted field validation using four Polarimetric Active Radar Calibrators (PARCs) deployed in the Inner Mongolia test site. The results showed that the amplitude error was less than 0.6 dB, phase mismatch was controlled within 5°, and cross-talk in the sidelobes was lower than −33 dB, demonstrating the system’s precise calibration capability and high signal-to-noise imaging performance [111]. Additionally, the LT-1 satellites provide stable multibaseline interferometric observations. Under a multibaseline configuration, the acquired data effectively capture forest canopy scattering characteristics, making LT-1 a critical data source for spaceborne forest height retrieval studies [112].

These advancements indicate that China’s PolInSAR technology and applications are rapidly evolving. Satellite platforms with multi-baseline and multi-polarization observation capabilities are providing novel remote sensing tools for forestry monitoring, agriculture, wetland protection, and related fields.

### 4.2. Geosynchronous Orbit

To address the issues of small imaging swath width and long revisit times of low-orbit SAR satellites in imaging the observation area, the Earth-Synchronous Synthetic Aperture Radar (GEO SAR) [113] emerged. By taking advantage of the orbital position of the Earth-Synchronous Orbit, the revisit period for the same location can be increased from the sub-day level of low-orbit SAR to the hour level, and the imaging swath width can be expanded from hundreds of kilometers of low-orbit SAR to thousands of kilometers. By improving the temporal resolution of the same observation scene, high-frame-rate SAR images, namely in the high-temporal mode, can be obtained, which extends the information to three dimensions. The additional temporal dimension provides more spatio-temporal degrees of freedom. It can provide strong support for the timely and effective implementation of disaster emergency responses.

The concept of high-orbit SAR satellites was first proposed in 1978 [114], and it remains a research hotspot to this day. NASA of the United States, Cranfield University of the United Kingdom [115], Politecnico di Milano of Italy [116], Vega Company of Russia, China Academy of Space Technology, and other research institutions [117,118,119] are all conducting research on high-orbit SAR.

**Figure 26:**
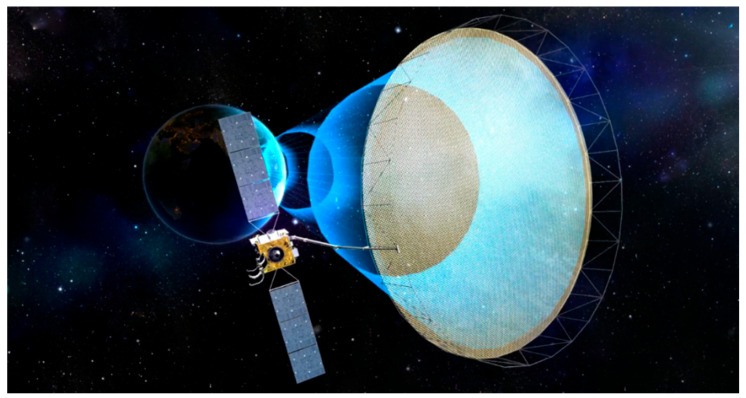
LT-4(01) Satellite configuration [image credit: CAST].

China was the first country to launch the GEO satellite, demonstrating a major breakthrough in high-orbit SAR technology. On 13 August 2023, China’s LT-4(01) satellite was launched, becoming the world’s first Earth-synchronous orbit SAR satellite [119], as shown in Figure 26. The synthetic aperture radar payload carried by the LT-4(01) satellite has advantages such as high resolution, wide coverage, multi-mode, and being lightweight. In addition to adopting synthetic aperture radar technology, this satellite has another feature, which is that its operating orbit is exceptionally high, about 40,000 km away from the Earth. The higher it flies, the farther it can see.

**Figure 27:**
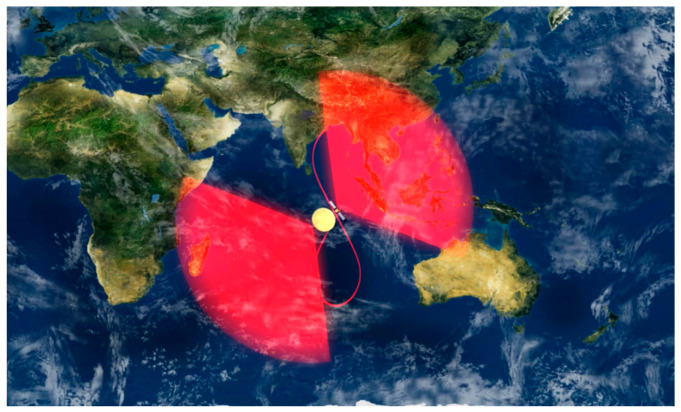
LT-4(01) Satellite work diagram [image credit: CAST].

**Figure 28:**
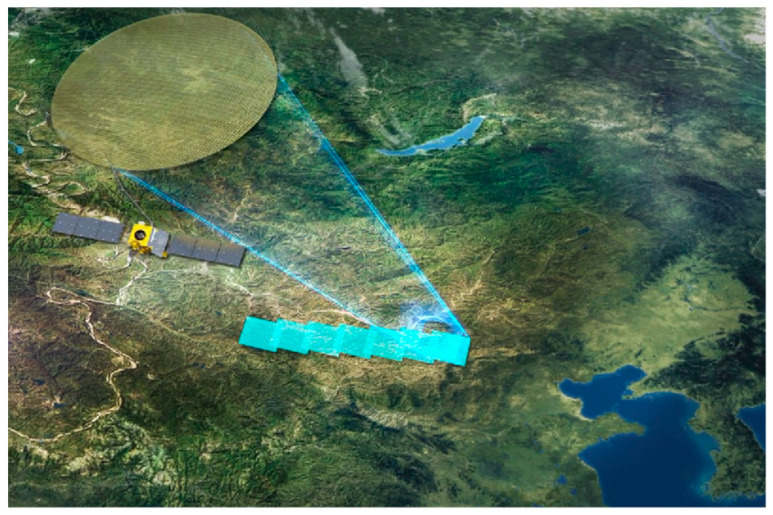
LT-4(01) Satellite scanning [image credit: CAST].

The LT-4(01) satellite operates in an inclined geosynchronous orbit at a distance of nearly 40,000 km from the Earth. Satellites operating at this orbit resemble a repeatedly drawn “8” shape in the sky, enabling relatively continuous observations of a fixed large area, as shown in Figure 27. The observation revisit period is short and the imaging swath is wide. Compared with low-orbit satellites and optical satellites, the LT-4(01) satellite combines the advantages of short high-orbit observation revisit periods and wide imaging swaths with the advantages of microwave observation that is not limited by weather conditions (all-weather) and not limited by lighting conditions (all-time). This can improve the recognition accuracy and efficiency of abnormal changes in disaster information and enhance the comprehensive prevention and control capabilities for natural disasters. In the development of the LT-4(01) satellite project, an innovative scheme of a large-aperture annular reflector antenna and phased array feed was proposed for the first time and implemented engineering-wise [120]. The imaging maximum swath of the high-orbit SAR satellite can reach 3000 km. It has the ability to conduct a full-basin observation of potential flood disasters in the Yangtze River and Yellow River basins. Operating in the scanning mode (50 m/3000 km), it can complete the coverage of the entire country within 3 days and form a national map, as shown in Figure 28. The same efficacy can only be achieved by networking five low-orbit SAR satellites.

### 4.3. Low-Earth Orbit

**Figure 29:**
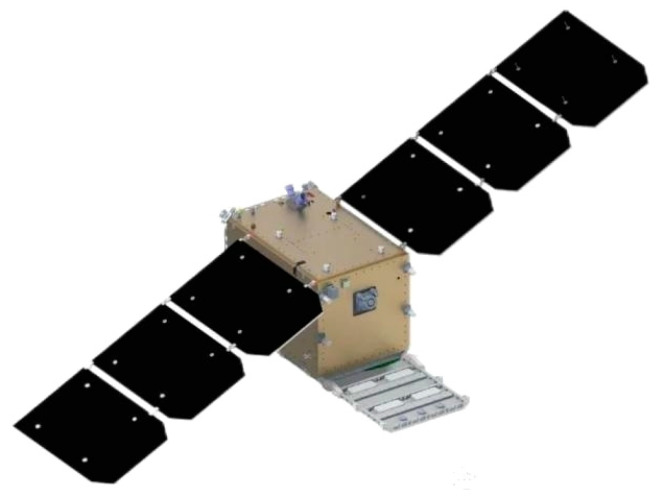
AIRSAT-08 low-earth-orbit SAR satellite [image credit: AIR].

The independently developed AIRSAT-08 marks a major breakthrough for China in the field of low-earth-orbit (LEO) SAR. AIRSAT-08 is the country’s first low-inclination ultra-low-orbit SAR remote sensing satellite, as shown in Figure 29. It was successfully launched in December 2024, marking a major leap from experimental verification to practical deployment in this technical domain. The lower orbital altitude significantly enhances radar echo strength and signal-to-noise ratio, enabling higher imaging resolution and stronger target identification capabilities under the same system parameters. In addition, the shorter revisit cycle and denser ground tracks of the LEO allow for the high-frequency coverage of observation areas, making it well suited for time-sensitive tasks. AIRSAT-08 is equipped with an X-band multi-polarization radar system and adopts a miniaturized design, with the onboard SAR payload weighing less than 80 kg. It supports multiple imaging modes including stripmap and spotlight, and achieves a maximum resolution of 0.1 m. On-orbit tests have demonstrated high-quality imaging and multi-polarization data acquisition, successfully validating the potential of low-orbit small SAR systems in high-resolution imaging, rapid revisit, and multi-dimensional observation [121].

In addition, the successful launch of the HJ-2-06 satellite further enhanced China’s deployment of low-Earth-orbit SAR systems. As a key component of the HJ-2 series, this satellite operates in LEO, together with two optical satellites, forming a “2 + 2” observation constellation. Its low-orbit configuration enables high revisit frequency and all-weather imaging, playing a vital role in disaster monitoring and emergency response. The improved temporal resolution and high-precision observation capability provided by LEO significantly strengthened data support for rapid response to emergencies in China [122].

### 4.4. HRWS

**Figure 30:**
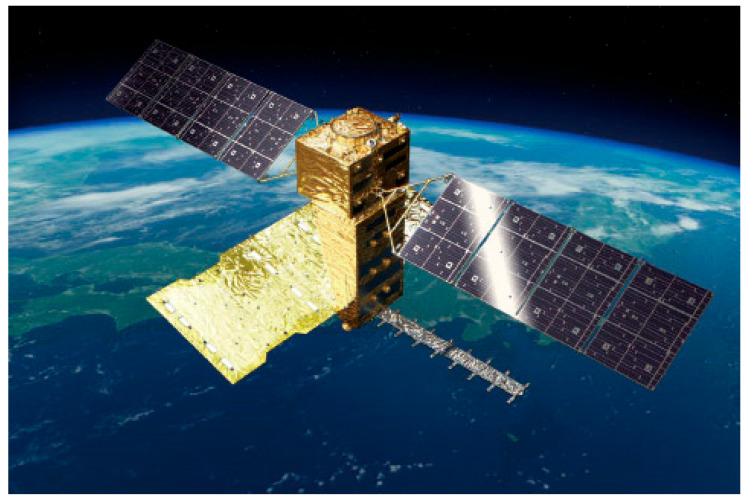
ALOS-4 satellite [image credit: JAXA].

Japan launched the Advanced Land Observing Satellite-4 (ALOS-4) on 1 July 2024. ALOS-4 in-orbit configuration as shown in Figure 30. The Japan Aerospace Exploration Agency (JAXA) released the first observation images captured by the L-band synthetic aperture radar (PALSAR-3) aboard the satellite from 15 to 17 July 2024 (Japan Standard Time). “ALOS-4” is the first radar satellite to implement digital beamforming technology on an artificial satellite. While maintaining the high spatial resolution of the previous “ALOS-2”, it has expanded the observation range by up to four times (200 km wide at a 3 m resolution) [123]. It is expected to contribute to disaster situation understanding, environmental observations such as forest distribution, and marine observations such as sea ice, together with “ALOS-2”.

In China, we are actively developing our own high-resolution wide-swath SAR systems. Zhou et al. designed and implemented an airborne X-band SAR system with 16 receiver channels in the elevation direction, supporting digital beamforming (DBF) to meet future HRWS imaging demands. The system operates with a 500 MHz bandwidth and adopts a Scan-On-Receive (SCORE) mode to expand the imaging swath while maintaining high resolution [124]. Through real flight experiments, the imaging performance was validated under realistic terrain conditions. The study addressed channel gain and phase mismatches by modeling and calibrating system errors. Results showed that the DBF technique significantly improved signal-to-noise ratio and ensured image consistency over a wide field of view. This work provides a technical foundation for future airborne and spaceborne HRWS SAR systems and offers an effective solution to the traditional trade-off between resolution and swath width.

### 4.5. Lunar and Deep Space Exploration

China has made significant strides in SAR technology for deep space exploration, advancing from technical groundwork to engineering applications. In the upcoming Phase IV of the Lunar Exploration Program, the Chang’e-7 (CE-7) orbiter will be equipped with a Pol-SAR for the first time, focusing on high-resolution imaging and water ice detection in the lunar polar regions. By analyzing polarimetric scattering characteristics, this payload can penetrate permanently shadowed areas to retrieve terrain and material information, addressing a technological gap in China’s deep space SAR capabilities and providing essential data for lunar resource exploration. In addition, the “China Fuyan” deep space radar system, developed by Beijing Institute of Technology, has entered its initial operational phase. The system consists of four distributed radar units, each with a 16 m aperture, and has successfully produced the first 3D radar image of a lunar impact crater, demonstrating the feasibility of distributed aperture radar imaging for deep space. By coordinating multiple small radar antennas, the system can actively image the Moon, near-Earth asteroids, and other planetary targets, enabling large-aperture observations of distant objects.

China’s deep space SAR research is expanding toward higher resolution, multi-platform coordination, and novel observation modes. The Pd-SAR system under development is expected to achieve 0.5 m resolution, enabling the detailed surface characterization of deep space targets. Proposed concepts such as the GCOLB-SAR system envision Earth observation from a stable lunar platform, offering ultra-wide swath coverage of up to 6500 km and providing a new paradigm for global change studies [125]. Concurrently, breakthroughs in multi-platform coordinated imaging and onboard real-time data processing are accelerating, addressing deep space data transmission bottlenecks and improving detection efficiency.

These developments demonstrate that China is actively advancing SAR technologies through independent innovation, enabling key applications in lunar base construction, planetary geological research, and deep space resource exploration.

### 4.6. Challenges

Although spaceborne InSAR theories and techniques have experienced nearly 60 years of development and have broken through many technical bottlenecks, they still face many technical difficulties and challenges. These are reflected in the following aspects.

(1) Multi-platform synchronization and multi-baseline keeping.

The multidimensional InSAR system consists of a radar transmitter and multiple space-separated radar receivers. This system has the characteristics of flexible observation baseline configuration and diverse observation angles, but also faces many technical challenges, including how to achieve high-precision synchronization between multiple platforms, how to achieve the accurate positioning of multi-phase centers, and how to achieve multi-baseline measurement and maintenance. In recent years, synchronization has become a key issue that needs to be urgently addressed in distributed spaceborne SAR systems. By analyzing the time synchronization error and the beam synchronization error, guidance can be provided for system design [126]. In order to improve the phase synchronization accuracy of LT-1 in the bistatic interferometry mode, a robust phase error estimation and compensation method was proposed, which utilized the Kalman filter to enhance the synchronization accuracy [127].

(2) Storage, transmission, and on-board processing of massive amounts of data.

While the InSAR system has many advantages, it generates a large amount of remote sensing data. In particular, the formation of an SAR system such as PIESAT-1 will generate an unprecedented amount of data and face huge challenges in storage, transmission, and processing. Efficient data compression, fast data transmission, and on-board real-time processing have become urgent problems to be solved in the industry. The on-board storage of InSAR data relies on the spaceborne solid-state storage system, which will greatly increase the cost of satellite development while increasing the storage capacity. InSAR data transmission is limited by the power and bandwidth of the on-board data transmission antenna and the design of the ground receiving station. The real-time transmission and storage of large volumes of data is still challenging. One example is to exploit the inherent parallelism of field-programmable gate arrays (FPGAs) [128]. Furthermore, the integration of artificial intelligence and machine learning techniques can be used to enhance data analysis and decision-making capabilities [129,130].

## 5. Conclusions

The InSAR technology progress and development trends introduced in this article mainly include repeat-pass interferometry and single-pass interferometry. In recent years, the number of SAR satellites launched has been increasing continuously, especially after commercial satellite companies joined the ranks of developing and launching SAR satellites. Moreover, due to the limitations of weather conditions, meaning that optical satellites cannot achieve all-weather earth observation, countries around the world need SAR satellites to fill the observation gaps of optical satellites.

Repeat-pass interferometry includes D-InSAR technology, PS-InSAR technology, and SBAS-InSAR technology, and its main application field is surface deformation observation. Traditional repeat-pass interferometry has a long revisit period, which means it cannot meet the timeliness requirements of emergency disaster reduction. Earth-synchronous orbit SAR satellites have a short revisit period and are not affected by weather and night images, and can achieve high-precision feedback for surface deformation products and timely warning.

Single-pass interferometry includes CT-InSAR and AT-InSAR technologies. CT-InSAR technology is mainly applied in DEM and DSM generation, as well as sea surface elevation measurement, while AT-InSAR technology is mainly applied in ocean surface current field measurement and moving target detection. Traditional single-baseline earth observation has a single dimension, and fixed baselines cannot adapt to different terrains and sea conditions. Development towards multi-satellite and multi-baseline capability is conducive to achieving multi-dimensional high-precision measurement.

We are proud to demonstrate that China has made substantial progress in the development of SAR systems, establishing a spaceborne SAR constellation that spans multiple frequency bands, imaging modes, and orbital configurations. Satellites such as the GF-3 series and the Land Exploration series have been widely applied in surface monitoring, disaster response, and deep space exploration. The systematic advancement of China’s SAR missions continues to enhance its influence and technological leadership in the global InSAR field.

## Figures and Tables

**Table 1:** Classification of typical surface deformation phenomena [4].

Geophysical Phenomenon	Process Classification				Spatial Scale/km	Deformation Scale/mm
	Instantaneous	Slow	Reversible	Irreversible		
Active volcano rises or sinks	— ^1^	✓ ^2^	✓	—	<20	<5
Volcanic eruption	✓	—	—	✓	<20	>50
Earthquake co-seismic deformation	✓	—	—	✓	50~100	>50
Deformation before and after earthquake	—	✓	—	✓	50~100	<5
Crustal fault movement	—	✓	—	✓	>20	<5
Surface settlement	—	✓	—	✓	0.5~20	1~20/a
Mining subsidence	✓	—	—	✓	0.1~10	1~100/d
Landslide (foreboding)	—	✓	—	✓	1~20	1
Landslide (eruption)	✓	—	—	✓	1~20	>1000

^1^ No. ^2^ Yes.

**Table 2:** Classification and D-InSAR technology.

Measurement	Precision Level	GNSS	D-InSAR
Spatial coverage	Discrete point	Discrete point	Surface covering
Accuracy	mm	mm	mm
Periodic velocity	Long and slow	Short and fast	Short and fast
Operating condition	According to the weather	All-weather	All-weather
Cost	High	higher	low

**Table 3:** Advantages and disadvantages of three technologies.

InSAR Technology	Advantages	Disadvantages	Accuracy
D-InSAR	Easy processing. Suitable for deformation monitoring in a small area.	Two or more high-quality SAR images are required. Low sensitivity. Not suitable for large-scale deformation monitoring and non-linear changes in surface deformation.	1~2 cm/a
SBAS-InSAR	High monitoring accuracy. Suitable for medium-range deformation monitoring.	A large amount of SAR data is required. Complex processing. Sensitive to the choice of baseline and requires caution.	2~5 mm/a
PS-InSAR	High precision and high sensitivity. Suitable for the monitoring of complex terrain and non-linear deformation. Stable surface targets such as buildings, telecommunication towers, etc., can be detected.	Complex processing. A large amount of SAR data is required. High requirements for the stability of ground objects.	0.5~1 mm/a

**Table 4:** Comparison of InSAR and other DEM acquisition techniques.

DEM Acquisition Technique	Coverage	DEM Accuracy
Ground	Local, large-scale mapping range	0.01~0.1 m
Airborne photogrammetry	Region	0.1~1 m
Airborne lidar	Region	0.5~2 m
InSAR	Regional to global	1~20 m
Shadow mapping	Regional to global	Slope ≤ 2°, 22 m
Stereo mapping	Region	10~100 m

**Table 5:** Comparison of digital terrain elevation data (DTED-2) and high-resolution terrain information (HRTI-3) specifications [4].

Requirement	Specification	DTED-2	HRTI-3
Relative vertical accuracy	90% linear point-to-point	12 m (slope < 20%)	2 m (slope < 20%)
	error over a 1° × 1° cell	15 m (slope > 20%)	4 m (slope > 20%)
Absolute vertical accuracy	90% linear error	18 m	10 m
Relative horizontal	90% circular error	15 m	3 m
Horizontal accuracy	90% circular error	23 m	10 m
Spatial resolution	independent pixels	30 m (1 arc sec at equator)	12 m (0.4 arc sec at equator)
